# Computational anatomy for studying use-dependant brain plasticity

**DOI:** 10.3389/fnhum.2014.00380

**Published:** 2014-06-27

**Authors:** Bogdan Draganski, Ferath Kherif, Antoine Lutti

**Affiliations:** ^1^LREN – Department for Clinical Neurosciences, CHUV, University of LausanneLausanne, Switzerland; ^2^Max Planck Institute for Human Cognitive and Brain SciencesLeipzig, Germany

**Keywords:** magnetic resonance imaging, computational anatomy, brain plasticity

## Abstract

In this article we provide a comprehensive literature review on the *in vivo* assessment of use-dependant brain structure changes in humans using magnetic resonance imaging (MRI) and computational anatomy. We highlight the recent findings in this field that allow the uncovering of the basic principles behind brain plasticity in light of the existing theoretical models at various scales of observation. Given the current lack of in-depth understanding of the neurobiological basis of brain structure changes we emphasize the necessity of a paradigm shift in the investigation and interpretation of use-dependent brain plasticity. Novel quantitative MRI acquisition techniques provide access to brain tissue microstructural properties (e.g., myelin, iron, and water content) *in-vivo*, thereby allowing unprecedented specific insights into the mechanisms underlying brain plasticity. These quantitative MRI techniques require novel methods for image processing and analysis of longitudinal data allowing for straightforward interpretation and causality inferences.

## Introduction

Groundbreaking research in the last two decades brought strong evidence of the lifelong capacity of the mature mammalian brain to respond to alterations in the environment or individual's homeostasis. Although the concept of brain plasticity was initially coined in a different neuroscientific context, we here adopt the definition of plasticity as an intrinsic brain property change driven mainly by a mismatch between existing functional supply and environmental demand or caused by primary changes in functional supply (Lovden et al., 2010). Brain plasticity can be studied at different scales—from molecular and cellular up to the systems level through the perspective of either brain structure and/or function. Building on pioneering studies in rodents based on *post mortem* assessment of experience-induced brain volume changes in rodents (Rosenzweig et al., 1962) and non-human primates (Wang et al., 1995) most recent *in vivo* works demonstrate at the cellular level the complex dynamics of use-dependant dendritic spine plasticity (for review Holtmaat and Svoboda, 2009) and at the systems level—the extent of cortical reorganization after lost of peripheral input (Flor et al., 1995).

In humans, the advancement of magnetic resonance imaging (MRI) and the development of sophisticated computer-based analytical methods capturing the complex patterns of brain shape, volume and surface characteristics—computational anatomy, opened new possibilities for *in vivo* studies of use-dependant plasticity. A steadily growing number of studies confirm the notion of a remodeling of brain anatomy, but fail to provide further insight into the underlying neurobiological processes. The main reason for this is the fact that current state-of-the-art imaging assessments of structural brain plasticity rely mostly on relative changes in gray matter volume, density, and cortical thickness derived from MRI data which are the result of multiple microstructural factors which cannot be disentangled.

Here we review the published literature on use-dependant plasticity of the adult human brain studied with MRI-based computational anatomy methods. The next section outlines novel theoretical frameworks for studying brain plasticity, followed by a section on the accumulated scientific evidence on use-dependant plasticity. Recent advances in MRI acquisition techniques are presented that offer promising prospects for the exploration of the neurobiological basis of brain plasticity. Considering the most recent reviews on the topic (Lovden et al., 2010; Zatorre et al., 2012; Thomas and Baker, 2013), we focus on the necessity for investigation and interpretation of training-induced brain anatomy changes based on the quantification of specific tissue properties rather than metrics such as gray matter volume or density which are rather loosely defined in neurobiological terms. Further, we stress the specific need for longitudinal studies with multiple time points of data acquisition before, during, and after behavioral intervention allowing for inferences about causality.

## Theoretical concepts

The concept of mutual link between modification of behavior and ability of the human brain for profound functional and structural reorganization throughout the whole lifespan is well established in modern neuroscience. However, after decades of research on the topic and empirical evidence for ongoing plasticity in the mature human brain we are still far away from understanding the basic neurobiological principles underlying plasticity (for review see Buonomano and Merzenich, 1998; Dayan and Cohen, 2011; Zatorre et al., 2012). An overwhelming number of studies at the cellular, synaptic, and systems level spanning a wide methodological spectrum confirm the notion of experience- induced interaction between complex processes embedded in a somewhat blurry theoretical framework. The conceptualization of use-dependant human brain plasticity across the lifespan turned out to be particularly challenging—both in terms of definition when differentiating between development and experience or learning as well as when looking for straightforward neurobiological interpretation of plasticity-associated brain MRI findings (Galvan, 2010; Zatorre et al., 2012; Draganski and Kherif, 2013; Thomas and Baker, 2013).

Paradoxically, one of the first neuroscientists suggesting a possible impact of exercise on the brain is Ramon y Cajal, better known for his view on the brain as fixed and immutable tissue (Ramon Y Cajal, 1894). More than 80 years later—in the late 1970s Paillard formulated Ramon y Cajal's assumptions in a theoretical framework postulating that plastic brain changes emerge only when experience-associated anatomy alterations have functional consequences rather than the opposite—when functional changes occur within established anatomical networks (Paillard, 1976). The framework acknowledged the importance of both structural brain connectivity and changes in its constitutive elements—the neurons, considering the fundamental principle of “lasting” plastic changes long after the triggering event (see also Will et al., 2008a,b).

More recently, this much needed theoretical framework was not only refined in terms of semantic precision (Lovden et al., 2010), but also amended with the clearer concept of an interaction between brain and environmental demand for plastic changes. The core of this concept is the notion of functional supply—demand mismatch triggering the system's ability for plastic change. Here, demand is used in the sense of requirement to perform a task whereas functional supply denotes the individual's capacity to function within a certain range of performance and is defined mainly by brain anatomy constraints. Supply—demand mismatch could either be due to primary changes in environmental demand—in the simple case adaptation to a new condition—or to primary changes in functional supply—injury after stroke or chronic degenerative process. The assumption of functional supply—demand mismatch requiring “lasting” plasticity changes is embedded in the frame of the “sluggishness” of the system's response, which integrates different manifestations of plasticity and the corresponding underlying physiological mechanisms—long lasting neuro-/ and angiogenesis vs. rapid long-term potentiation (LTP)—based modulation of dendritic spines dynamics (Lovden et al., 2010).

The evolution of these current theoretical concepts provides a flexible framework to integrate recent findings of use-dependant brain plasticity at various scales and across different analytical techniques. Inherent part of these concepts is the notion of causal pathways, which, translated in the field of computational anatomy, motivates the need for longitudinal studies with multiple data acquisition time points allowing for inferences about causality. The newly proposed model (Lovden et al., 2010) addresses in a holistic way pertinent questions about causes, consequences and dynamics of use-associated brain plasticity leading to new ideas for future research.

## Brain plasticity—macrostructural changes

The emergence of structural and functional MRI has provided a unique opportunity for non-invasive *in vivo* investigation of plasticity-associated changes across the whole-brain. According to the experimental design, studies on brain plasticity can be divided in two types—cross-sectional studies with a single data acquisition time point and longitudinal studies with multiple time points of data acquisition. While inferences from cross-sectional studies remain at the descriptive level of correlation analysis, longitudinal studies bring the potential for revealing how behavioral changes result from the temporal dynamic of interaction between brain regions.

### Cross-sectional studies of brain plasticity

Cross-sectional computational anatomy studies investigate the correlation between brain structural features and training-/learning abilities by comparing cohorts of experts and non-experts in a particular field. Milestone previous studies reported associations between e.g., navigational experience and volume of the posterior hippocampus (Maguire et al., 2000), musical proficiency and volume increase in motor and auditory areas (Gaser and Schlaug, 2003; Hyde et al., 2009). The supposition here was that local brain volume changes would represent the use-dependant plasticity of a particular region or system implicated in the specific function of interest—assumption supported by the correlation between magnitude of change and individual performance.

Identically to computational anatomy studies on gray matter volume, voxel-based analysis of water diffusion indices from diffusion-weighted data demonstrated differences associated with piano practice (Bengtsson et al., 2005), bimanual coordination skills (Johansen-Berg et al., 2007) and grammar learning abilities (Floel et al., 2009). The major limitation of correlation analyses using brain imaging data obtained at a single time point is the inability to infer causality and to distinguish between the independent use-associated effects on brain structure from the impact of environmental and pre-existing intra-individual factors (Draganski and Kherif, 2013).

### Longitudinal studies of brain plasticity

Addressing the major limitation of cross-sectional studies in use-dependant plasticity—inability to distinguish between cause and consequence (i.e., “nature” vs. “nurture”), we aimed to infer causality using a prospective study design with multiple data acquisition time-points (Draganski et al., 2004). Led by the assumptions that neurogenesis drives use-dependant brain structure changes and considering findings reporting a 3 months period needed for differentiation of a pluripotent stem cell to mature neuron (Cummings et al., 2005), we carried out a longitudinal study involving juggling training. Young volunteers were scanned at three time points—before starting to learn how to juggle, 3 months after, followed by another 3 months period with restriction from juggling. We observed transient gray matter increases in the extra-striate motion specific area hMT/V5 bilaterally and in the left inferior parietal cortex. The demonstrated regional gray matter changes reversed nearly to baseline paralleled by decrease in juggling performance at the third time point. No significant structural changes were detected in the control group where data was acquired at the same time points as in the intervention group.

Following the same neurobiological assumption of neurogenesis underlying use-dependent plasticity in the adult human brain we monitored morphometric changes related to memory and learning over three time points each 3 months apart (Draganski et al., 2006). The behavioral intervention consisted of intensive preparation for the German preliminary medical exam including both oral and written exams in biology, chemistry, biochemistry, physics, social sciences, psychology, human anatomy, and physiology, which demanded high level of encoding, retrieval, and content recall. Our findings demonstrated differential effects of learning regarding dynamic temporal characteristics on cortical structures. Besides the predicted changes in medial temporal lobe structures where hippocampal and parahippocampal gray matter expanded continuously through the three time points we detected initial gray matter increase in posterior parietal cortex between the first and second time point without further change toward the third time point.

Subsequent longitudinal studies confirmed the notion of use-dependent anatomy remodeling both in the brain gray and white matter (Golestani and Pallier, 2007; Scholz et al., 2009; Taubert et al., 2010; Bezzola et al., 2011; Herholz and Zatorre, 2012; Meyer et al., 2012; Sagi et al., 2012; Steele et al., 2013). Neuroimaging studies on juggling, acquisition of auditory skills, balance, and musical training and spatial navigation showed strong effects of behaviorally relevant interventions on local brain volume and white matter microstructure. White matter plasticity studies reported differential directionality of use-dependent changes in water diffusion indices—fractional anisotropy and mean diffusivity, most likely due to local differences in underlying white matter architecture.

Clearly, many principled questions on the topic of use-dependant plasticity studied with computational anatomy remain unanswered. Our assumptions on the temporal dynamics and linearity of exercise-associated brain anatomy changes next to the often-neglected impact of intra-individual variability (Kanai and Rees, 2011) are important points to be considered in future studies. The potential interaction between these factors and the effects of ageing motivate the investigation of training-induced plasticity across different age groups and particularly the interaction with ongoing developmental processes during puberty or progressive neurodegeneration associated with ageing and brain disorders (Sehm et al., 2014). Finally, we also cannot ignore the fact that neuroimaging techniques—while overcoming the limitations of animal studies regarding invasiveness and restricted volume of investigation—suffer from a coarse spatial resolution, which only allows for inferences at the macroscopic level.

## Brain plasticity—cellular mechanisms

Brain plasticity can be studied at different scales ranging from sub-cellular to macroscopic brain systems level, which is mirrored in the abundance of invasive methods to investigate *in vivo* structural and functional aspects of brain remodeling. The dynamic link between use-dependant modulation of behavior and brain structure requires specific anatomical changes enabling optimal information processing. Theoretically, the underlying physiological mechanisms could be due to alterations in the number and morphological properties of neuronal and glial cells, synaptic connectivity, axons, and myelin as well as angiogenesis (Zatorre et al., 2012).

At the macroscopic level animal studies conducted in the 1960s demonstrated correlations between enriched environment and increases in cerebral cortex volume and total brain weight (Rosenzweig et al., 1962; Bennett et al., 1964). The experimental setting consisted of frequent changes and rearrangement of toys in the animals' cage building on the Hebbian idea of complex enrichment (Hebb, 1949). Follow-up studies in rodents in enriched environment reported experience-associated brain plasticity changes in the range of 3-20% (Black et al., 1990; Anderson, 2011) in line with observations in birds (Clayton and Krebs, 1994). In the following decades neurobiology research focused on investigations at the sub-cellular level using sophisticated analytical tools (e.g., immuno-histochemistry, quantitative electron microscopy, two photon laser microscopy, opto-physiological recordings) to capture *in vivo* or *post mortem* the modulation of synaptic strength and the turnover of synaptic and dendritic spines (for review Holtmaat and Svoboda, 2009). The Hebbian postulates of LTP, long-term depression and the spike-timing dependant plasticity were considered as basic physiological mechanisms underlying synaptic remodeling (Raymond, 2007). More recent studies revealed the plasticity-dependant role of glia associated with synapses (for review Haber and Murai, 2006; Henneberger et al., 2010). The supposition here was that use-dependant synaptic plasticity mediated by glutamatergic synaptic transmission and guided by neuronal activity represents the structural basis of learning and memory (for review Holtmaat and Svoboda, 2009). Converging evidence supported the notion that use-dependant functional remodeling is not only the result of connectivity changes associated with structural plasticity at the synaptic level (Kleim et al., 2007). Adding another layer of complexity, studies attributed an important role to the myelinated perineuronal nets as structural and functional “brakes” limiting morphological changes associated with use-dependant plasticity in the mature brain (Pizzorusso et al., 2002). Along these lines, combined *in vivo* MRI imaging and *ex vivo* histological studies in mice subjected to different versions of the water maze task demonstrated convincingly not only the anatomical specificity of the reported training- induced brain structure changes, but also strong evidence for associated axonal growth rather than changes in neuronal cell size or number (Lerch et al., 2011).

A strong argument against the assumption that neocortical neurogenesis outside the hippocampal dentate gyrus could underlie use-dependant changes in the mature human brain comes from a *post mortem* study measuring the integration of human DNA with (14)C isotopes generated by nuclear bomb tests during the Cold War. The main finding of this study is that neocortical neurogenesis outside the hippocampal dentate gyrus is restricted to the developmental period rather than a lifelong property of the human brain (Bhardwaj et al., 2006). Using the same (14)C isotopes assessment method the same group confirmed the functional relevance of hippocampal neurogenesis in the mature human brain with estimated 1.75% annual turnover of hippocampal neurons (Spalding et al., 2013). Nevertheless, interpretations about specific role of neurogenesis in use-dependant brain plasticity could still be based on the assumption that newly generated hippocampal neurons can migrate to distant anatomical sites (Uchida et al., 2000; Pereira et al., 2007).

The main drawbacks of the abovementioned studies are their invasiveness and their limited observations of single synapses or restricted areas of single neurons. Aiming to bridge structure and function at the theoretical level, recently developed computational models started implementing rules for anatomical modifications at the cellular level to look for dynamic network property changes over time (Butz et al., 2008).

## Computational anatomy of brain plasticity—interpretational issues

In the field of imaging neuroscience the introduction of state-of-the-art mathematical algorithms (i.e., computational anatomy) for automated data analysis in the spatial and temporal domains enabled unbiased feature reductions and statistical parametric mapping in standardized space. Despite the overwhelming variability of existing software solutions for the computation of volume, surface, and shape characteristics of the brain rely on the very same basic principles of data processing and statistical analysis. Although computational anatomy studies on use-dependant brain plasticity raised hopes to answer pertinent questions about neurobiological processes underlying the remodeling of brain anatomy, only few attempts have been made to validate measures of relative changes in gray matter volume, density and cortical thickness derived from MRI data with “gold standard” histology assessment.

### Gray matter changes

The most widespread computational anatomy technique—voxel-based morphometry—VBM (Ashburner, 2009), provides automated brain volume and cortical thickness estimation for statistical inference on a population of interest. VBM includes an iterative algorithm combining voxel-by-voxel classification of each participant's MRI data into different tissue classes based on class-specific priors with the spatial registration to a common anatomical space. Following this step, the brain tissue classes are low-pass filtered by convolution with an isotropic Gaussian kernel (i.e., smoothing) to enter classical mass-univariate or multivariate statistical analyses. The inherent divergence of VBM based on MRI images from histological analysis of brain tissue properties is confirmed by a recent study, which failed to show any correlation between tissue classification probabilities estimated with computational anatomy algorithms and histological measures of neuronal density (Eriksson et al., 2009).

Automated estimation of voxel-based cortical thickness uses gray matter, white matter and CSF tissue partitions created in the classification step of VBM to extract cortical gray matter boundaries (Hutton et al., 2009). Subsequently, the voxel-based cortical thickness maps are registered to a common standardized space using the deformation fields applied for gray matter warping. The surface-based method for cortical thickness estimation implemented in Freesurfer (http://surfer.nmr.mgh.harvard.edu) relies on atlas information to detect the boundaries of gray matter followed by projection of the thickness values on a surface mesh (Dale et al., 1999; Fischl et al., 1999). Estimation of cortical thickness is assumed to provide more straightforward interpretation of computational anatomy results but is strongly affected by spatial and temporal changes in the brain tissue properties underlying MRI contrast changes (Salat et al., 2009; Lutti et al., 2014).

Despite a steadily growing number of computational anatomy studies a detailed insight into the neurobiological processes underlying use-dependant brain plasticity is still lacking. One reason for this is that most acquired MRI data is a mixed contribution of multiple brain tissue properties (e.g., myelin, iron, and water protons bound to macromolecules; Tofts, 2003) which cannot be disentangled at the analysis stage. Also, the specific effects of exercise and brain development and ageing on the microstructural properties affected by brain plasticity remain largely unknown and could lead to significant tissue classification bias and misinterpretation of the detected volume and cortical thickness changes. Additionally, commonly acquired MRI data is known to be severely affected by scanner-related effects leading to increased inter-scanner variability and reduced sensitivity which might explain some of the discrepancies observed across study sites (Weiskopf et al., 2013).

### White matter changes

The investigation of use-dependant brain white matter changes using computational anatomy and T1-weighted MRI images is reduced to only few VBM studies from the past (Golestani et al., 2002; Golestani and Pallier, 2007). With the emergence of diffusion-weighted imaging research focused on plasticity-associated white matter microstructure changes by inferring directionality and magnitude of water diffusion (i.e., fractional anisotropy and mean diffusivity) (Pierpaoli and Basser, 1996). However, the currently existing computational anatomy analytical frameworks are not fully adapted for the analysis of parameter data (e.g., fractional anisotropy, mean diffusivity value etc.), which hampers the straightforward interpretation of results. Exemplified by VBM in the SPM framework, statistical inferences about local gray matter volume changes are based on tissue probability estimates derived from the MR signal in a Bayesian framework using anatomically informed tissue priors (see above). Subsequent neurobiological interpretation is enabled by adjustments for linear and non-linear interpolation effects of spatial transformation, which preserve the total signal. In the case of fractional anisotropy or mean diffusivity parameters this framework is not readily applicable without modifications. Recent work provided potential solution for quantitative multi-parameter data, however the concept should be validated for the special case of DWI derived parameters (Draganski et al., 2011). In more general terms, the lack of specificity of the underlying biophysical model carries the risk of overinterpreting inferences made on the basis of diffusion-weighted data (Jones et al., 2013). The theoretical and empirical resolution of these issues is subject of recent attempts to correlate *in vivo* obtained diffusion-tensor derived indices with histology results (Concha et al., 2010; Sagi et al., 2012), resolve interpretational ambiguities (Douaud et al., 2011) and propose novel biophysical models (Zhang et al., 2012).

## Current methodological advances and outlook

Addressing the specificity limitations of commonly used MRI anatomical data, novel multi-parameter quantitative mapping protocols disentangle the contribution of each MRI parameter (e.g., T1, T2,…) to the acquired signal. Quantification of these MRI parameters, which correlate with brain tissue microstructural properties such as myelin, iron and water content allows for an unprecedented insight into the mechanisms underlying brain plasticity. This represents a paradigm shift in how we estimate and analyse brain anatomy features, which can be used to relate plasticity-associated brain tissue property alterations to changes in behavior and to estimate their specific impact on the currently used gray matter volume, density and cortical thickness. A tipping point for future advances in understanding the principles of human brain plasticity will be the creation of causal generative models based on longitudinal studies with multiple data time points acquired before, during, and after behavioral intervention. Finally, the biophysical mechanisms linking tissue microstructure and the MRI signal are still under investigation. Accurate modeling of these mechanisms will be essential to produce estimates of the microstructural properties relevant in brain plasticity studies from the quantitative MRI data. The development of biophysical models will require validation, most preferably in the form of correlation studies with “gold standard” histology assessment.

### Quantitative structural MRI

Quantitative structural brain imaging allows differentiating between plasticity-associated tissue property changes and local brain volume/density or cortical thickness changes. Our approach includes whole-brain multi-parameter mapping at high resolution (Helms et al., 2008; Helms and Dechent, 2009) correction for radio frequency transmit inhomogeneities (Lutti et al., 2010, 2012), and an established analytical framework—voxel-based quantification (VBQ) (Draganski et al., 2011). The ability of this approach to deliver robust and sensitive biomarkers of brain tissue properties has been demonstrated in a number or recent studies (Dick et al., 2013; Sereno et al., 2013; Lutti et al., 2014). Using VBQ we showed parameter-specific distribution patterns in healthy ageing and suggested a biophysical interpretation, which corroborates with histological studies showing age-dependant iron accumulation and rate of de−/remyelination (Draganski et al., 2011).

### Statistical inferences on causality

The investigation of causal relationships between use-dependant plasticity changes in the mature human brain has not been approached systematically yet. Researchers suggested a combination of brain stimulation techniques (transcranial magnetic stimulation—TMS and transcranial direct current stimulation—TDCS) and computational anatomy studies to lend support to causality assumptions (Kanai and Rees, 2011). Similarly, attempts to combine neural activity with brain anatomy measurements to study brain plasticity remained at the descriptive level due to a lack of prior knowledge about causal and temporal dynamics (Ilg et al., 2008; Haier et al., 2009). Future analytical strategies could capitalize on novel methods in a Bayesian generative model framework that provides the causal link between behavior, neural activity and tissue property changes. The explanatory and predictive power of the model could be tested in a data-driven approach integrating temporal and spatial scales of imaging data in parallel with histology assessment to facilitate biological interpretation.

### Comparative histological studies

Despite the steadily growing number of longitudinal computational anatomy studies reporting use-dependant brain structure changes, there has been no attempt to date to link macrostuctural morphometric findings with microstructural information (for review (Fields, 2009). We still face a very limited number of systematic comparative studies in animals demonstrating use-dependant regional volume changes assessed with established computational anatomy methods to correlate these with *post mortem* histological findings at higher spatial resolution. Though scientifically highly advantageous, the concept of looking for brain plasticity correlates at the microscopic level has its limitations—results from animal models cannot readily be extrapolated to humans and many cognitive processes cannot be studied in animals. There are also practical limitations such as shrinking of the specimen due to fixation, artifacts of staining or difficult localization of investigation/recording sites (Sincich and Horton, 2005), which hamper the comparison of histological results with computational anatomy studies.

In conclusion, the overwhelming number of computational anatomy studies on use-dependant brain plasticity has provided major contributions to our understanding of the characteristic spatial and temporal patterns of brain structure changes at the macroscopic scale. First animal studies applying in parallel computational anatomy methods and histological investigation provide important findings at unprecedented fine granularity. Newly emerging MRI acquisition techniques hold promising prospects that might allow the detection for the first time of the brain tissue microstructural changes associated with use-dependant plasticity beyond the vague neurobiological observation of concomitant volume or density changes. Not only will this advance our scientific understanding of brain plasticity, it will also provide an empirical basis for the creation of reliable non-invasive biomarker for the rehabilitation progress after brain function loss. A generative model capturing spatial and temporal characteristic of use-dependant changes could help in unraveling causal pathways between exercise-induced behavioral changes and remodeling of brain structure to make an important contribution to our scientific understanding of brain function and dysfunction.

### Conflict of interest statement

The authors declare that the research was conducted in the absence of any commercial or financial relationships that could be construed as a potential conflict of interest.
